# Fungal volatile organic compounds: mechanisms involved in their sensing and dynamic communication with plants

**DOI:** 10.3389/fpls.2023.1257098

**Published:** 2023-09-22

**Authors:** Rosario Razo-Belmán, Yesenia Ithaí Ángeles-López, Luis Fernando García-Ortega, Claudia Geraldine León-Ramírez, Lucila Ortiz-Castellanos, Houlin Yu, Domingo Martínez-Soto

**Affiliations:** ^1^ Departamento de Alimentos, División de Ciencias de la Vida, Universidad de Guanajuato, Irapuato, Guanajuato, Mexico; ^2^ Science and Math, Arkansas State University Queretaro, Colón, Querétaro, Mexico; ^3^ Departamento de Ingeniería Genética, Centro de Investigación y de Estudios Avanzados del IPN, Unidad Irapuato, Irapuato, Mexico; ^4^ Department of Biochemistry and Molecular Biology, University of Massachusetts Amherst, Amherst, MA, United States; ^5^ Departamento de Microbiología, Centro de Investigación Científica y de Educación Superior de Ensenada, Ensenada, Baja California, Mexico

**Keywords:** fungal volatiles, VOCs plant sensing, differential plant responses, beneficial or detrimental fungi, application of fungal VOCs

## Abstract

Microbial volatile organic compounds (MVOCs) are mixtures of gas-phase hydrophobic carbon-based molecules produced by microorganisms such as bacteria and fungi. They can act as airborne signals sensed by plants being crucial players in triggering signaling cascades influencing their secondary metabolism, development, and growth. The role of fungal volatile organic compounds (FVOCs) from beneficial or detrimental species to influence the physiology and priming effect of plants has been well studied. However, the plants mechanisms to discern between FVOCs from friend or foe remains significantly understudied. Under this outlook, we present an overview of the VOCs produced by plant-associate fungal species, with a particular focus on the challenges faced in VOCs research: *i*) understanding how plants could perceive FVOCs, *ii*) investigating the differential responses of plants to VOCs from beneficial or detrimental fungal strains, and finally, *iii*) exploring practical aspects related to the collection of VOCs and their eco-friendly application in agriculture.

## 1 Introduction

Microbial volatile organic compounds (MVOCs) are a wide diversity of hydrophobic carbon-based molecules produced by the primary and secondary metabolism of microorganisms such as fungi and bacteria. They are evaporable and can easily travel through the air over long distances (El Jaddaoui et al., 2023). This characteristic is important because it allows MVOCs to act as airborne signals that can be detected by aboveground and underground microorganisms, animals, and plants, influencing their growth and behavior (Diehl et al., 2023; El Jaddaoui et al., 2023; Kandasamy et al., 2023). MVOCs are considered long-distance messengers in intra- and inter-kingdom ecological interactions.

Classically, volatile organic compounds (VOCs) are described as small odorous compounds with a low molecular weight (< 300 Da), low boiling point, and high vapor pressure under normal conditions (0.1 kPa at 20°C). These characteristics allow their easy spreading through the atmosphere and even soil (El Jaddaoui et al., 2023). Interestingly, numerous studies have demonstrated the crucial role of VOCs emitted by plants in establishing and influencing interactions with other living organisms (Angeles-Lopez et al., 2018; Razo-Belman et al., 2018). Moreover, it has been demonstrated that MVOCs produced by beneficial or pathogenic microorganisms have effects on plant physiology, metabolism, development, and priming (Sarkar and Sadhukhan, 2023). These findings strongly suggest that VOCs influence the plant-fungal interactions.

Fungal volatile organic compounds (FVOCs) production has been described for species of all fungal phyla (Devi et al., 2020; Guo et al., 2021a). Notably, mycorrhizal fungi and mycoparasitic and soil-borne ascomycetes species such as *Trichoderma asperellum* and *Trichoderma harzianum*, are known to induce a priming effect on plants through the production of specific FVOCs, which in turn activates genes involved in pathogen response pathways (Wonglom et al., 2020; Esparza-Reynoso et al., 2021; Sarkar and Sadhukhan, 2023). In this review, we briefly discuss the characteristics and roles of the FVOCs produced by fungi with different lifestyles associated with plants, the mechanisms of plants for sensing FVOCs, and the molecular responses of the plants to the sensed FVOCs. Also, we overview the aspects related to the VOCs collection and their eco-friendly application in agriculture.

## 2 Biosynthesis and chemical characteristics of the FVOCs

FVOCs are synthesized from molecules and precursors generated throughout the carbon metabolic pathways, for example: acetyl-coA, erythrose 4-phosphate, phosphoenolpyruvate, and pyruvate. Their synthesis also requires fundamental elements such as sulfur and nitrogen commonly found in living organisms (Kaddes et al., 2019). In fungi, the biochemical pathways potentially involved in FVOCs biosynthesis are the mevalonate pathway (Coppola et al., 2019), the shikimate pathway (Achimón et al., 2022), the polyketide biosynthetic pathway (Weisskopf et al., 2021), and the fatty acid-derived oxylipin pathway (Kaddes et al., 2019). For example, the mevalonate pathway is prominently related to FVOCs production as it directly participates in enzymatic reactions involved in the synthesis of terpenes and terpenoids (Kaddes et al., 2019; Abdulsalam et al., 2021). Recently, sesquiterpenes have gained attention due to their significant roles in fungal-plant interactions, and fungal-fungal interactions (Bruisson et al., 2023; Costa almeida et al., 2023). In *Trichoderma atroviride*, a model organism of beneficial fungi for plants, the *Lox1* gene is involved in the biosynthesis of pentyl-α-pyrone (6-PP), a FVOC with plant growth-promoting and antifungal activities (Moreno-Ruiz et al., 2020; Speckbacher et al., 2020). Also, in *Trichoderma*, the FVOCs production is affected by defects in the secondary metabolism as a result from the NADPH oxidases deletion (*Nox*) (Villalobos-Escobedo et al., 2020), which are essential genes encoding proteins with roles in several biosynthetic pathways of FVOCs.

An important characteristic of FVOCs is their high diversity of chemical structures: aliphatic alcohols, cycloalkanes, aldehydes, esters, ketones, benzenoids, naphthalene derivatives, terpenoids, etc. (Camarena-Pozos et al., 2021; Guo et al., 2021a; Shahi et al., 2022). This high diversity was recently corroborated by the analysis of the VOC profiles of forty-three fungal species, where 256 FVOCs were identified. Interestingly, the distinct patterns of FVOCs were correlated with the different fungal lifestyles (Guo et al., 2021a). Moreover, this wide range of chemical structures enables FVOCs to possess several biological activities, such as antimicrobial, insecticidal, and plant growth-promotion.

In summary, the biosynthesis of FVOCs appears to be a holistic phenomenon involving multiple biochemical pathways, including carbon metabolism and several biosynthetic pathways. Undoubtedly, the study and identification of key regulatory genes controlling the biosynthetic pathways of FVOCs, merit further efforts of dedicated research.

## 3 The plant sensing of volatile organic compounds

The research of MVOCs sensing by plants provides valuable insights into their diverse roles in plant defense, communication, and environmental interactions. Several studies have explored the effects of MVOCs on plant-microbe interactions. For example, research on VOCs of *Trichoderma asperelloides* strain PSU-P1 revealed their ability to inhibit the growth of phytopathogens, promote plant growth, and activate defense responses in *Arabidopsis thaliana* (Phoka et al., 2020). Similarly, VOCs produced by *T. asperellum* strain T1 were found to inhibit fungal growth, induce defense responses, and enhance growth in lettuce, with the analysis identifying 22 volatile compounds contributing to these effects (Wonglom et al., 2020).

**Figure 1:**
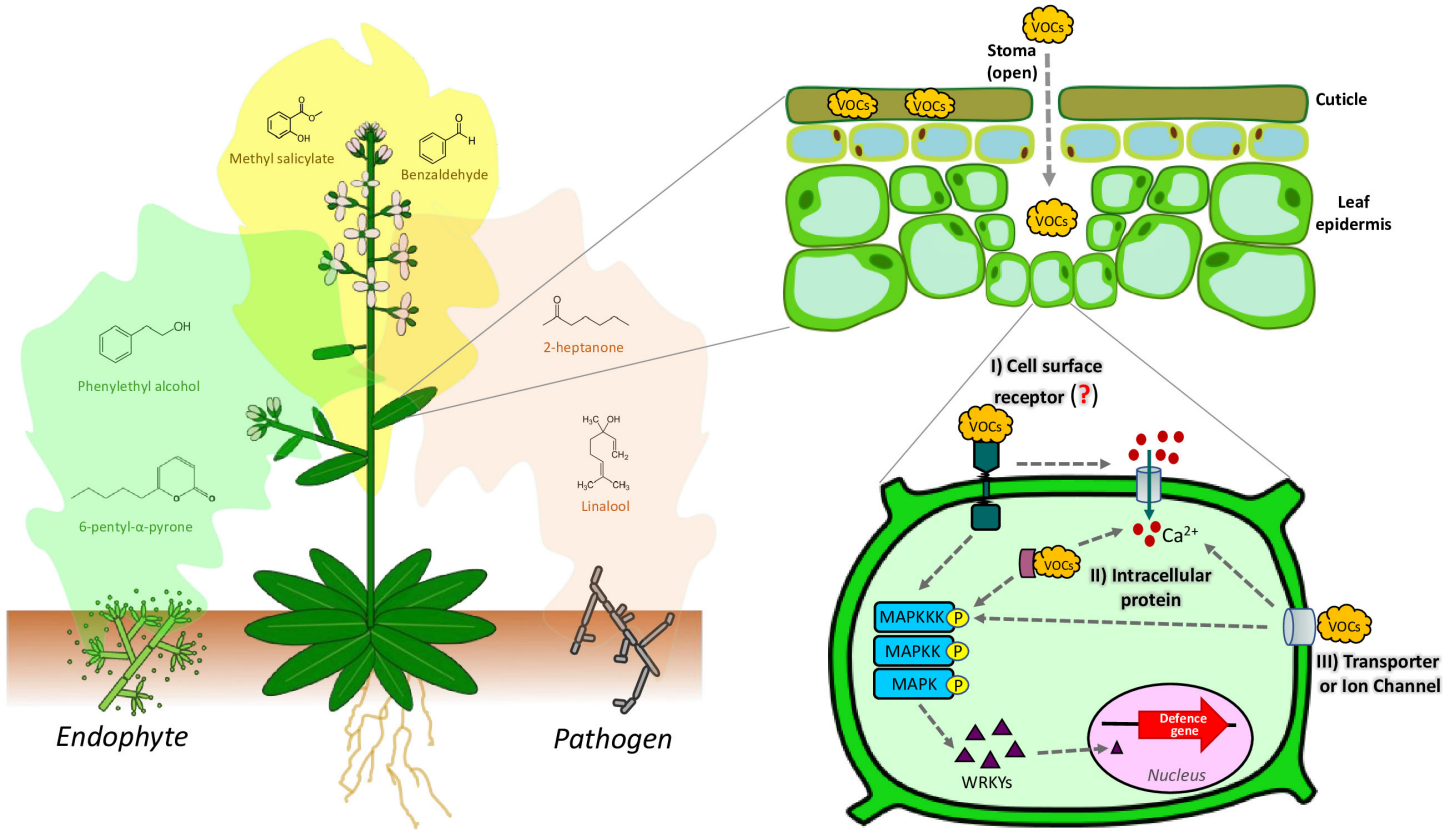
General representation of the sensing and responses of plants to the FVOCs. Notice the sequestration of VOCs by the cuticular waxes and the passage of VOCs through the stomata to reach the extracellular space. So far, it has been hypothesized that plants perceive FVOCs in three ways: *I*) Cell surface receptors, *II*) intracellular proteins, and *III*) volatile transporters or ion channels. FVOCs trigger a cascade of defense, including calcium influx and the activation of Mitogen-Activated Protein Kinases (MAPKs), which in turn activate the WRKY transcription factor. WRKY is involved in the expression of genes related to defense mechanisms. Differential plant responses to FVOCs profiles from beneficial or detrimental fungi have been suggested. However, this hypothesis has been little studied.

Beyond fungal VOCs, other studies have shed light on the impacts of non-fungal VOCs (such as from plants themself) on plant growth, defense responses, and inter-organism communication. For example, recent studies have shown the important role of the stomata in the perception of VOCs (Aguirre et al., 2023), and the importance of cuticular waxes to sequester exogenous VOCs (Camacho-coronel et al., 2020), since VOCs need access to the cell plasma membrane or intracellular compartments to trigger the various responses like the plant defense response (**Figure 1**). Thereafter,VOCs are internalized to the plant cells through the stomata and cuticular wax layer, and then they move through the cell wall and air space, where we hypothesize they interact with non-specific lipid transfer proteins (nsLTPs) (Liao et al., 2023). However, the membrane receptors involved in VOCs detection are unknown. It has been hypothesized that VOCs could be recognized by specific binding receptors or cross the cell membrane through specific transporters or ion channels which transfer the signal to the nucleus by signal transduction pathways such as the mitogen-activated protein kinase (MAPK) pathways (Wang and Erbo, 2022) (**Figure 1**). Based on this, it could be suggested that some mechanisms are being conserved in plants to sense VOCs. Interestingly, plants also emit VOCs in response to various stimuli, triggering signaling cascades that activate defense responses (**Figure 1**). For instance, when plants are attacked by fungal pathogens or colonized by beneficial fungi, they release microbial-induced plant volatiles that prime neighboring plants for defense (Brilli et al., 2019; Sarkar and Sadhukhan, 2023).

The emission of VOCs by plants serves as a means of communication and provides valuable information about their physiological status, influencing primary and secondary metabolism, growth, and reproduction of the receiver plant (Brosset and Blande, 2022). Notably, specific VOCs released by tomato plants in response to herbivory can trigger in neighboring plants a rapid change in plasma membrane potential and increase in calcium fluxes into the cytosol induced by the reactive oxygen species (ROS) production (Fincheira et al., 2021). Similarly, it has been suggested that MVOCs influence ROS production, calcium influx, and nitric oxide (NO) signaling; which in turn can be associated with the MAPK signaling pathways (Wang et al., 2017; Sharifi and Ryu, 2018), influencing the regulation defense mechanisms, photosynthesis, nutrients balance, metabolism, hormone crosstalk, etc. (Fincheira et al., 2021) (**Figure 1**). However, the mechanisms of VOCs perception in plants have not been clarified. Different authors suggest that the role of specific VOCs receptors may be similar to odorant binding proteins (OBPs), as seen in animals (Loreto and D’Auria, 2022). However, the OBPs originally described to bind to methyl salicylate and methyl jasmonate are not very selective (Giordano et al., 2021). Recently, it was described that non-specific lipid transfer proteins (nsLTPs) localized in the cell-wall, facilitates VOCs emission (Liao et al., 2023). This allows to hypothesize that VOCs uptake occurs at the inverse of the emission, where the nsLTPs participate in the transport of VOCs from the environment into the cell.

In summary, research on the sensing of VOCs by plants provides valuable insights into the potential sensing mechanisms of FVOCs by plants, and their roles in defense, communication, fitness, and environmental interactions. Understanding these mechanisms enhances our knowledge of plant-microbe interactions and holds implications for crop protection and ecosystem management.

## 4 Can plants differentiate FVOCs from beneficial or pathogenic fungi?

The capacity of plants to perceive and respond to environmental cues is essential for survival. Plants respond to volatiles from different origins, such as herbivores, microbes, and even other plants. However, it is important to highlight that the FVOCs role depends on their concentration, some FVOCs are toxic at high concentrations, but the same FVOCs contribute to inducing plant resistance at low concentrations. Thus, they may act as positive or negative mechanism regulators of plant defenses (Wang and Erb, 2022).

Plants can perceive MVOCs from a long distance and use them to prime themselves and better respond to other microorganisms representing potential negative agents affecting plant growth, fitness, and development (Weisskopf et al., 2021). VOCs emitted by beneficial and pathogenic microorganisms can promote plant growth and improve plant tolerance to biotic and abiotic stresses (Velásquez et al., 2020). However, some FVOCs emitted by pathogenic fungi are classified as phytotoxins negatively affecting plant growth, for instance: 1-octen-3-ol, *trans*-2-octenal, 1-hexanol, 3-octanone, 3-methyl-1-butanol and 2-phenylethanol (Werner et al., 2016). The compounds 1-octen-3-ol and *trans*-2-octenal inhibit root and cotyledon leaf growth, and induce bleaching of the seedlings in *A. thaliana* by H_2_O_2_ production (Ferreira et al., 2023; Wang et al., 2023). Another example of FVOCs with negative effects on plants are those emitted by *Fusarium culmorum* and *Cochliobolus sativus*, which modify the emission of barley VOCs from roots during their interaction, affecting the possibility to prime neighborhood plants. Moreover, FVOCs from these pathogens decrease the length of roots and leaf surface area (Jamil et al., 2020; Duc et al., 2022).

On the other hand, lettuce plants respond to FVOCs emitted by the endophyte fungus *T. asperellum* by increasing the production and activity of enzymes for fungal cell-wall degrading as chitinase and β-1,3-glucanase enhanced their resistance against pathogenic fungi like *Corynespora cassiicola* and *Curvularia aeria* (Wonglom et al., 2020). Also, those FVOCs increase the number of leaves and roots, plant biomass, and chlorophyll content. All these findings together suggest that plants can differentially respond to beneficial or harmful microorganisms. However, it remains unclear whether plants can recognize and/or distinguish between FVOCs from friend or foe, since differential colonization between beneficial and pathogenic fungi has been observed (Guo et al., 2021b; Martínez-Soto et al., 2023). To solve this concern, Moisan et al. (2019) tested if plants can distinguish between FVOCs from pathogenic and non-pathogenic soil-borne fungi. Under their hypothesis, FVOCs can be used to determine specificity for plants, which means, the volatiles of pathogenic fungi could be perceived as a ‘warning’, allowing plants to be prepared for the attack of a potential antagonist. Whereas volatiles of non-pathogenic fungi could be perceived as a ‘message’ by plants and facilitate their contact with a potential mutualist. However, they found that *A. thaliana* plants did not discriminate between FVOCs from pathogenic and non-pathogenic fungi, based on plant phenotypic responses. The Arabidopsis response to FVOCs from both fungi was the same, promoting plant growth and flowering after the exposition of FVOCs.

## 5 Roles of the FVOCs on plants

Plants and fungi are intimately associated partners under mutualistic and antagonistic relationships (Guo et al., 2021b; Martínez-Soto et al., 2023). FVOCs play an essential role in establishing these associations, they can affect or promote plant development and their resistance against pathogens (Hu et al., 2021). Recently, a differential plant perception of FVOCs profiles emitted by beneficial or detrimental fungi has been observed, suggesting a differential response of plants to microorganisms with different lifestyles (pathogens versus endophytes) and with unique profiles of FVOCs (Moisan et al., 2019).

Moreover, FVOCs can trigger plant signaling pathways which can crosstalk with other signal transduction pathways, enhancing or suppressing the plant defense and the plant innate immunity (Erb, 2018). For instance, 1-octen-3-ol induces the expression of the jasmonic acid (JA)/ethylene -dependent genes such as allene oxide synthase (*AOS*), hydroperoxide lyase (*HPL*), the wounding-dependent defense genes *PDF1.2*, and pathogenesis-related protein *PR-3*. The activation of all these genes enhances the resistance of *A. thaliana* against *Botrytis cinerea* (Kishimoto et al., 2007). However, 1-octen-3-ol can also act as a detrimental volatile compound depending on the concentration (see the above subtitle).

The methyl benzoate emitted by *Ampelomyces* sp. and *Cladosporium* sp., two non-phytopathogenic fungi, induce the systemic resistance in *A. thaliana* against *Pseudomonas syringae* by the whole and partial activation of JA-signaling and salicylic acid (SA)-signaling pathways respectively (Naznin et al., 2014). Additionally, methyl benzoate induces the expression of genes related to the plant defensin gene *PDF1.2*, the transcription factor *MYC2*, the vegetative storage gene *VSP2*, and the pathogenesis-related protein *PR-1* gene (Naznin et al., 2014). Other examples of FVOCs emitted by non-pathogenic fungi are 2-methyl-1-butanol, 2-pentylfuran, acetic acid, and 6-pentyl-2H-pyran-2-one; emitted by *T. asperelloides*, those activate the Arabidopsis defense responses by the activation of the peroxidases, chitinases, and β-1,3-glucanase production (Phoka et al., 2020). Similarly, 6-pentyl-2H-pyran-2-one increased in grapevine leaf the accumulation of callose, the expression of defense-related gene *PR-2*, and the expression of the hypersensitive response-related genes as a defense mechanism against the pathogens *Plasmopara viticola* (Lazazzara et al., 2021).

Finally, as an example of plant responses to FVOCs emitted by phytopathogenic fungi, it has been described the re-localization of Arabidopsis resources and their growth and accelerated reproductive development caused by FVOCs of the root pathogen *Rhizoctonia solani*. Under this pathogen-plant interaction, the Arabidopsis root growth and development is induced by up-regulation of genes involved in auxin signaling and down-regulation of genes related to ethylene (ET) and JA signaling pathways (Cordovez et al., 2017; Kidd et al., 2021).

## 6 Application of the FVOCs

The interest in exploring the potential biotechnological applications of FVOCs has increased in the last decade. So far, around 250 FVOCs have been reported, many of them have potential applications such as biofuel production, flavor or fragrance additives, and biosensors of plant diseases. In addition, several studies have proved the efficiency of FVOCs as fungicides, insecticides, bactericides, nematocidal, resistance inducers to abiotic and biotic stresses, plant-growth promoters, as well as biomedical applications (Inamdar et al., 2020; Razo-Belman and Ozuna, 2023).

**Figure 2:**
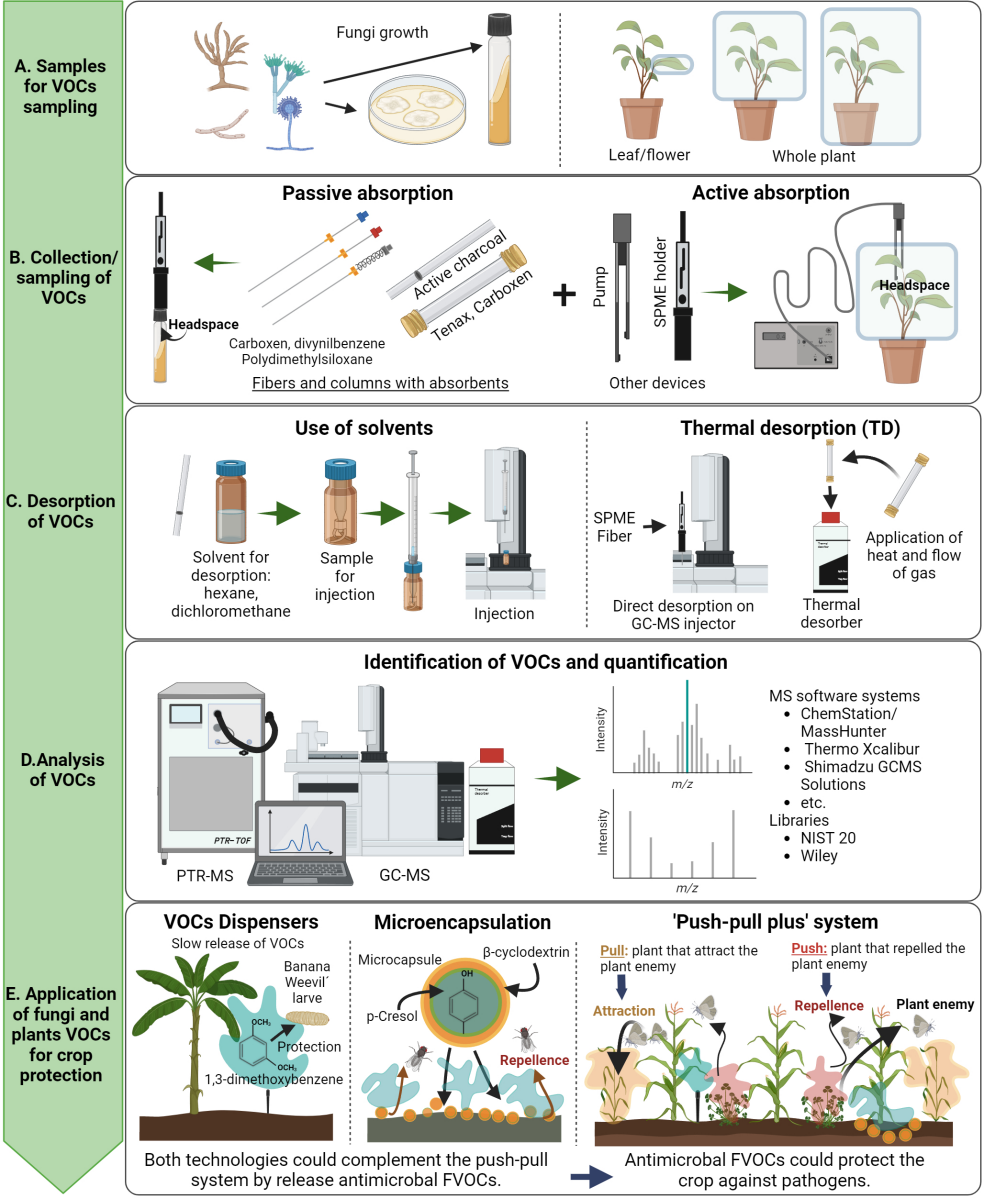
General overview of the steps for collection, analysis, and applications of FVOCs and plant VOCs on the field. Notice from top to bottom the description of the process for collection, analyzing, and application of VOCs. The whole process has been divided in five main steps: **(A)** Types of samples for VOCs sampling; **(B)** Collection and sampling of VOCs; **(C)** Desorption of VOCs; **(D)** Analysis of VOCS; and **(E)** Application of fungi and plants VOCs. Figure created with BioRender.com.

So far, the analysis of FVOCs has been performed by two techniques, the Gas Chromatography coupled with Mass Spectrometry (GC-MS) and Proton Transfer Reaction coupled with Mass Spectrometry (PTR-MS). Both identify and quantify gaseous molecules in the air and track VOCs released by different organisms, such as plants, insects, and microorganisms (Majchrzak et al., 2018). For the analysis of FVOCs and plant VOCs, we can follow the steps described in **Figure 2**: *i*) Collection of samples which can be performed by passive or active absorption using fibers and columns with absorbents or pumps respectively (**Figures 2A, B**), *ii*) Desorption of VOCs using solvents or thermal desorption (**Figure 2C**), and *iii*) Analysis of VOCs which includes quantification and identification of VOCs using pure standards compounds and specialized libraries respectively (**Figure 2D**). Interestingly, pure or mixtures of VOCs can be released in the field for crop protection. VOCs can be applied on the field with technologies such as dispensers, microencapsulation, and in the case of plant VOCs, using the “Push-Pull” system (**Figure 2E**) (Razo-Belman and Ozuna, 2023).

Dispensers allow the slow application of VOCs in field or greenhouse conditions for prolonged periods. For example, the FVOC 1,3-dimethoxybenzene produced by the entomopathogenic fungi *Beauveria bassiana* and *Metarhizium robertsii* has been applied using open dispensers in commercial banana crops to repel larvae of the Banana weevil *Cosmopolites sordidus* (Lozano-Soria et al., 2023) (**Figure 2E**). Microencapsulation is a technology in which a functional barrier of nanomaterials, packs the VOCs of interest. This method maintains the properties of VOCs, preventing its loss by chemical or physical reactions (Bakry et al., 2016; Kłosowska et al., 2023). For example, the microencapsulation of the FVOCs, β-ocimene, phenol, p-cresol, and indole, uses the heptasaccharide β-cyclodextrin as a barrier, when used as biocontrol of *Musca domestica* (Alonso et. al., 2021) (**Figure 2E**). Finally, the ‘Push–Pull’ system has been successfully implemented in Africa to control lepidopteran pests (Pickett et al., 2014). The purpose of this technology is keeping out herbivorous insects away from the crop of interest, by combining the crop with other plants that repel or attract a specific herbivore (Stenberg et al., 2015; Yi et al., 2019). For instance, maize is intercropped with *Desmodium uncinatum* as the repellent plant, and *Pennisetum purpureum* as the attractive plant in the crop-field border (**Figure 2E**).

The challenge for the implementation of the “Push-Pull” system with other crops is finding the pair of plants that repel or attract the same pest in combination with a crop of interest (Yi et al., 2019). Another possible limitation of the “Push-Pull” system is the lack of protection against pathogen microorganisms since the system protects plants against insect pests only (Pickett et al., 2014). Taking this into consideration, we hypothesize that a “Push-Pull plus” system involving the simultaneous application of the canonical push-pull system, with dispensers of FVOCs or microencapsulated FVOCs, could be implemented on the field (**Figure 2E**) preventing both, the crop attack against herbivore insects as well as improving the plant defense mechanisms and plant fitness.

## 7 Conclusions and perspectives

The role of MVOCs in plant growth promoting, plant development, and plant protection against herbivorous insects and phytopathogenic microorganisms such as fungi has been well established. However, there are still challenges in understanding fungal-plant interactions, including the role of the signal transduction pathways, the plant receptors for sensing the diverse range of FVOCs, and determining whether plants can differentiate between FVOCs emitted by beneficial and harmful fungi. This last one is particularly important considering the distinct plant colonization behavior exhibited by endophytic and pathogenic fungi, and the different phenotypic responses of plants to these fungi. Additionally, more efforts are needed to identify the master regulators of the biosynthetic pathways of FVOCs. Understanding these regulatory mechanisms can provide valuable insights into the biosynthesis of FVOCs and their specific functions in fungal-plant interactions.

Another important challenge is to develop suitable biotechnological strategies for the application of VOCs on crops in agriculture. Especially, considering the ease with which VOCs evaporate. Fortunately, with the vast fungal and plant genomic information available, pangenomic analysis can help to identify conserved or specie-specific volatiles, and the molecular mechanisms involved in the production and sensing of VOCs. Additionally, advancements in nanotechnology, such as microencapsulation or nanoparticles, offer potential solutions for protecting and controlling the release of VOCs in the environment. These technological advancements and their combination with others already established, such as the “Push-Pull” system or dispensers, can aid in the practical application of VOCs in agricultural settings, even under the variable environmental conditions caused by climate change. Addressing these challenges will contribute to a deeper understanding of the intricate fungal-plant interactions mediated by VOCs and pave the way for the development of innovative strategies for sustainable agriculture.

## Author contributions

RR-B: Conceptualization, Investigation, Visualization, Writing – original draft, Writing – review & editing. YÁ-L: Conceptualization, Investigation, Visualization, Writing – original draft, Writing – review & editing. LG-O: Writing – original draft. CL-R: Writing – original draft. LO-C: Writing – original draft. HY: Writing – original draft. DM-S: Conceptualization, Investigation, Project administration, Supervision, Visualization, Writing – original draft, Writing – review & editing.
